# Supplementary material for: Targeting epigenetic regulators: In-silico discovery of natural inhibitors against histone demethylase KDM4C
Source: PLoS One. 2026 Jan 8;21(1):e0340107. doi: 10.1371/journal.pone.0340107 (PMC12782423; doi:10.1371/journal.pone.0340107)
Supplement: Attachment — Submitted filename: Targeting.pdf [file pone.0340107.s003.pdf]

Manuscript title: Targeting epigenetic regulators: In-silico discovery of natural inhibitors against histone demethylase KDM4C

Reviewer's Report

The study presents a computational exploration to identify natural polyphenolic inhibitors of histone demethylase **KDM4C**, a known oncogenic epigenetic regulator. Through molecular docking, molecular dynamics (MD) simulations, MM-GBSA free energy calculations, and ADMET profiling, the authors identify **pectolinarin** and **compound 202** as potential inhibitors. Overall, the research addresses a relevant therapeutic target and employs well-established computational tools, yet several areas need refinement to enhance reproducibility, clarity, and interpretive depth.

The **study design** is appropriate for a computational investigation and aligns with its objectives. However, the manuscript would benefit from clearer justification of the screening library's selection and inclusion criteria. The authors effectively integrate multiple in-silico methods, but they rely exclusively on proprietary software (Schrödinger Suite) without comparative validation using open-source tools. Including a brief commentary or supplementary comparison using **AutoDock Vina** or **PyRx** would increase accessibility and reproducibility.

The **methods** are generally detailed but should include explicit parameters for MD simulations, such as temperature, pressure control, and solvation model specifics, to facilitate repetition by independent researchers. Statistical measures for convergence and error handling in energy calculations should be clarified. The use of **MM-GBSA** is appropriate, though an explanation of frame selection and sampling criteria would improve transparency.

The **results** are presented clearly, and the figures effectively illustrate molecular interactions and stability parameters. The statistical treatment of uncertainties is acceptable for a computational study, though quantitative descriptors (e.g., RMSD averages with standard deviations) should be added to strengthen reliability. The **discussion** would benefit from a more critical appraisal of the limitations of in-silico predictions and from suggesting concrete experimental validation steps, such as enzymatic inhibition or cell-based assays, to support translation. The conclusions are supported by the evidence presented but are somewhat overstated; the authors should moderate claims about "therapeutic potential" until experimental validation is performed.

No ethical concerns are present, as no human or animal data are involved. The **authors have declared no conflicts of interest**, and the **funding statement** is transparent. A

standard **similarity check** is still advised to confirm originality, given that the study covers a well-established domain. The figures appear original and free from manipulation, but all structural models and docking visualizations should be explicitly cited as generated from **PDB ID: 5KR7**.

Overall, the presentation of the work is coherent and logically structured, with minor language inconsistencies (e.g., "pectolaniarin" vs. "pectolinarin") already corrected in the revised version. The manuscript aligns with *PLOS ONE*'s standards for methodological rigor and data transparency, provided the minor revisions below are incorporated.

Recommendation: Accept with Minor Revisions

Essential revisions required for acceptance:

- Add reproducibility commentary comparing Schrödinger results with open-source alternatives.
- Include a concise "Methodological Limitations" or "Future Work" paragraph in the conclusion.
- Quantify statistical variation in RMSD and energy data.
- Ensure consistent terminology and reference formatting.
- Provide access details for the screened compound dataset and parameters used for molecular docking.

Ethics and Integrity Summary:
No ethical violations or data integrity issues identified. Figures are original and appropriately labeled. The data availability statement satisfies *PLOS ONE* standards.
