# Supplementary material for: Targeting epigenetic regulators: In-silico discovery of natural inhibitors against histone demethylase KDM4C
Source: PLoS One. 2026 Jan 8;21(1):e0340107. doi: 10.1371/journal.pone.0340107 (PMC12782423; doi:10.1371/journal.pone.0340107)
Supplement: Attachment — Submitted filename: Rebuttal_MKedit.docx [file pone.0340107.s002.docx]

**Reviewer Comment and answers**

The paper titled “Targeting epigenetic regulators: In-silico discovery of natural inhibitors against histone demethylase KDM4C” looks at how to use computer-based methods to find natural compounds that could block KDM4C, an epigenetic regulator linked to several cancers. The study integrates virtual screening, molecular docking, molecular dynamics simulations, and MM-GBSA free energy calculations to evaluate compound binding and stability. The research focuses on a current and significant topic in cancer treatment by suggesting that computer-identified natural compounds, which have good ADMET profiles, could be used as potential epigenetic therapies. Overall, is a relevant and detailed study with translational potential, though some issues related to presentation, clarity, and critical analysis need attention.

We thank the reviewer for the valuable suggestions and the overall positive assessment of our work. We thank the reviewer for valuable suggestions. We have modified the manuscript and incorporated suggestion into the manuscript. The answers to the questions are provided below.

The issues are:

1. Fix the typographical inconsistencies (e.g., “pectolaniarin” vs. “pectolinarin”).

Thank you for pointing out the typographical inconsistency. We have carefully reviewed the manuscript and corrected all instances of the misspelling to ensure uniformity and accuracy throughout the text.

2. The work heavily relies on proprietary software (Schrödinger suite), and it does not discuss reproducibility using open-source tools. Consider including brief commentary on reproducibility or comparison with open-source methods such as AutoDock Vina.

The present research is primarily based on the proprietary Schrödinger suite, which provides robust and highly accurate computational tools for molecular docking, dynamics, and visualization. Nevertheless, to improve reproducibility and accessibility, it is crucial to recognize the possibility of replicating essential elements of the study using open-source alternatives. Tools like AutoDock Vina, PyRx, and Open Babel can be utilized for molecular docking studies, yielding reasonably accurate and reproducible results. Although these tools may not possess some of the advanced features or automation found in Schrödinger, they are extensively used and validated within the scientific community. We also tested manually docked the top compound with the Autodock Vina and found the values very similar to the obtained values from the Schrodinger but not mentioned in this manuscript.

3. The discussion overemphasizes docking scores without accompanying in vitro or in vivo validation, which limits the impact. Add a clearer discussion of the limitations of in silico predictions and the need for experimental confirmation.

We acknowledge the reviewer’s valuable comment regarding the overreliance on docking scores without experimental validation. Indeed, while molecular docking serves as a critical initial step in virtual screening by predicting the binding affinity and orientation of ligands within the target protein’s active site, it is not sufficient alone to confirm the efficacy or biological relevance of the identified hits. Docking scores are influenced by static structural assumptions and do not account for protein flexibility or solvent effects, which can significantly impact ligand binding in a physiological environment. To address this limitation and strengthen the reliability of our findings, we employed molecular dynamics (MD) simulations to evaluate the stability and behavior of the protein–ligand complexes under dynamic conditions. This allowed us to observe the conformational adaptability and consistent binding interactions of the top ten lead compounds, as well as the control molecule, over time. The results revealed stable binding poses and key interactions that were preserved during the 200-ns simulations, suggesting potential biological relevance. Furthermore, to complement the MD simulations, we performed MM/GBSA binding free energy calculations to estimate the thermodynamic favorability of each ligand–protein complex. These calculations provided quantitative insight into the strength of interactions and helped identify residues with significant energetic contributions through per-residue decomposition analysis. These in silico methods together offer a multi-parametric justification of lead selection beyond docking scores alone.

4. The conclusion largely restates the abstract and lacks critical evaluation of study limitations. Use the conclusion to reflect on the limitations of computational-only studies and propose concrete next steps (e.g., planned wet-lab assays).

In our revised conclusion, we have explicitly addressed the inherent limitations of computational-only studies namely, the reliance on in silico predictions without experimental validation, and the potential divergence between computational results and actual biological outcomes. To advance this research, we propose conducting targeted wet-lab assays, such as enzymatic inhibition and cellular activity tests, to validate the computationally identified candidates. These experimental studies are crucial for confirming the therapeutic efficacy and biological relevance of our findings, and will form the basis of our next research phase.

We trust these revisions offer a more balanced perspective and provide a clear roadmap for future work, in accordance with the reviewer’s recommendation.

5. Maintain uniform formatting, with some journal titles abbreviated and others not. A uniform referencing style is needed.

We did the formatting as recommended.

Overall, this is a solid and methodologically thorough computational study that identifies natural inhibitors of KDM4C with potential anticancer activity. Although the findings show promise, the manuscript could benefit from a stronger emphasis on the limitations of computational predictions and a more critical discussion. I recommend minor revisions to improve clarity, scientific rigor, and presentation.

We implemented the suggestion in revised manuscript.
