# Supplementary material for: Targeting epigenetic regulators: In-silico discovery of natural inhibitors against histone demethylase KDM4C
Source: PLoS One. 2026 Jan 8;21(1):e0340107. doi: 10.1371/journal.pone.0340107 (PMC12782423; doi:10.1371/journal.pone.0340107)
Supplement: Attachment — Submitted filename: Epigenetic regulators review.docx [file pone.0340107.s004.docx]

Dear Authors,

I thank you for submiotting your manuscript, ''Targeting epigenetic regulators: In-silico discovery of natural inhibitors against histone demethylase KDM4C'' for review. I have completed a thorough review of your work, and noted that your study addresses an important topic in cancer research, leveraging advanced computational methods to identify potential KDM4C inhibitors from natural compounds. However, in its current state, your manuscript has multiple areas that have affected its overall clarity and scientific precision.

1. Consistency and Clarity Issues in Reporting Your Results:

i. Refer to the RMSD values,

I noticed remarkable inconsistencies between the data presented in Figure 3A and your textual descriptions of RMSD values. Example; The figure shows values fluctuating around '1-1.5 Å' for Pectolinarin while the text statement maintained RMSD values at below '0.5 Å'. Similarly, comparing the figure and textual RMSD values for compound 202 and the control complex, there are also notable discrepancies.

Recommendtion; Reconciled these discrepancies to enhance the manuscript's scientific accuracy.

ii. Refer to Figure 5A,

You had labeled this as showing "H-bond" on its Y-axis, however in the text, you referred to it as RMSD and Rg.

Recommendation: Kindly review and clarify the labeling and referencing mismatch.

iii. Refer to the Results section.

Regarding ADMET evaluation, you stated, "The selected top natural compound adheres to Lipinski’s Rule of Five..."

Comment/Recommendation; It's unclear which specific "top natural compound" you refer to, since multiple candidates were identified. You need to clarify if this applies to a any specific compounds or generally to Pectolinarin and compound 202.

2. Captions or Labels for Figures and Tables

Refer to Figures 6, 7, 8:

It seems you used a generic introductory sentence for each of these figures, "Molecular dynamics analysis of KDM4C interactions with the control complex is presented as follows". However, reviewing these figures, I noticed the figures in addition to the control, actually show interactions for Pectolinarin, compound 202, 707, Orientin, and Oroxin.

Recommendation: This gives a view of a misrepresentation or at best an error in reading of the fndings. You may wish to review this.

3. Typos.

I noticed multiple typographycal errors, where words were either overly spaced or not spaced at all. There was also an inconsistent spelling for "Pectolinarin", sometimes it was spelt as "Pectolaniarin", at other times as "pectolarinin". Please identify all these and effect a correction to enhance clarity.
