# Supplementary material for: Targeting epigenetic regulators: In-silico discovery of natural inhibitors against histone demethylase KDM4C
Source: PLoS One. 2026 Jan 8;21(1):e0340107. doi: 10.1371/journal.pone.0340107 (PMC12782423; doi:10.1371/journal.pone.0340107)
Supplement: Attachment — Submitted filename: Response to reviewer.docx [file pone.0340107.s005.docx]

**­­­­­­Reviewer Comment and answers**

I thank you for submitting your manuscript, ''Targeting epigenetic regulators: In-silico discovery of natural inhibitors against histone demethylase KDM4C'' for review. I have completed a thorough review of your work, and noted that your study addresses an important topic in cancer research, leveraging advanced computational methods to identify potential KDM4C inhibitors from natural compounds. However, in its current state, your manuscript has multiple areas that have affected its overall clarity and scientific precision.

We thank the reviewer for the valuable suggestions and the overall positive assessment of our work. We have modified the manuscript and incorporated suggestion into the manuscript. The answers to the questions are provided below.

1. Consistency and Clarity Issues in Reporting Your Results:

i. Refer to the RMSD values,

I noticed remarkable inconsistencies between the data presented in Figure 3A and your textual descriptions of RMSD values. Example; The figure shows values fluctuating around '1-1.5 Å' for Pectolinarin while the text statement maintained RMSD values at below '0.5 Å'. Similarly, comparing the figure and textual RMSD values for compound 202 and the control complex, there are also notable discrepancies.

Recommendation; Reconciled these discrepancies to enhance the manuscript's scientific accuracy.

**Answer:** We sincerely appreciate the reviewer's insightful comments regarding the figure presentations. We have carefully revised the manuscript to rectify these issues. The descriptions for Figures 3A (protein RMSD) and 3B (ligand RMSD within the KDM4C complex) have been made more precise throughout the text. Additionally, we have removed the duplicated image (previously Figure 4A) and consolidated the data presentation. We apologize for the oversights and thank the reviewer for helping us improve the quality of our work.

ii. Refer to Figure 5A,

You had labeled this as showing "H-bond" on its Y-axis, however in the text; you referred to it as RMSD and Rg.

Recommendation: Kindly review and clarify the labeling and referencing mismatch.

**Answer:** We thank the reviewer for this insight and have revised the manuscript accordingly.

iii. Refer to the Results section.

Regarding ADMET evaluation, you stated, "The selected top natural compound adheres to Lipinski’s Rule of Five..."

Comment/Recommendation; It's unclear which specific "top natural compound" you refer to, since multiple candidates were identified. You need to clarify if this applies to a any specific compounds or generally to Pectolinarin and compound 202.

Answer: We thank the reviewer for this valuable insight. The suggestion has been incorporated into the revised manuscript.

2. Captions or Labels for Figures and Tables

Refer to Figures 6, 7, 8:

It seems you used a generic introductory sentence for each of these figures, "Molecular dynamics analysis of KDM4C interactions with the control complex is presented as follows". However, reviewing these figures, I noticed the figures in addition to the control, actually show interactions for Pectolinarin, compound 202, 707, Orientin, and Oroxin.

Recommendation: This gives a view of a misrepresentation or at best an error in reading of the findings. You may wish to review this.

Answer: We acknowledge the oversight and have implemented the necessary revision in the updated manuscript.

3. Typos.

I noticed multiple typographycal errors, where words were either overly spaced or not spaced at all. There was also an inconsistent spelling for "Pectolinarin", sometimes it was spelt as "Pectolaniarin", at other times as "pectolarinin". Please identify all these and effect a correction to enhance clarity.

Answer: We appreciate the reviewer's keen attention to detail in identifying the typographical inconsistency. We have performed a comprehensive review and have unified the terminology in the revised manuscript to ensure accuracy.
