# Supplementary material for: Targeting epigenetic regulators: In-silico discovery of natural inhibitors against histone demethylase KDM4C
Source: PLoS One. 2026 Jan 8;21(1):e0340107. doi: 10.1371/journal.pone.0340107 (PMC12782423; doi:10.1371/journal.pone.0340107)
Supplement: Attachment — Submitted filename: Reviewers response.docx [file pone.0340107.s006.docx]

Review Comments to the Author

Please use the space provided to explain your answers to the questions above. You may also include additional comments for the author, including concerns about dual publication, research ethics, or publication ethics. (Please upload your review as an attachment if it exceeds 20,000 characters)

**Reviewer #3:** The manuscript presents a well-structured and technically sound in-silico investigation aimed at identifying natural compounds as potential KDM4C inhibitors. The computational methods including virtual screening, molecular docking, MD simulations, and MM-GBSA analysis are appropriate and rigorously applied. The results are clearly presented and support the conclusions drawn.

We thank the reviewers for the valuable suggestions and the overall positive assessment of our work. We have modified the manuscript and incorporated suggestion into the manuscript. The answers to the questions are provided below.

To strengthen the manuscript, I recommend the following revisions:

(1) Please simplify or break down some long paragraphs to improve readability, example, The MD simulation revealed stable ligand–protein interaction, and the RMSD remained within acceptable limits throughout the 100 ns simulation, indicating complex stability. Furthermore, hydrogen bonding analysis suggested persistent interactions, confirming the ligand’s potential as an inhibitor, and the MM-GBSA values were favorable, demonstrating strong binding affinity.”

this can be more specific and broken down to this;

The MD simulation revealed stable ligand–protein interactions. The RMSD remained within acceptable limits throughout the 100 ns simulation, indicating overall stability of the complex. Additionally, hydrogen bonding analysis showed persistent interactions. Together with favorable MM-GBSA values, these results suggest strong binding affinity.

Answer: We have simplified the text in the revised manuscript.

(2) Clarify certain methodological details such as ligand library sourcing and the rationale for specific simulation parameters to improve reproducibility;

Answer: We have incorporated the library information in the revised manuscript.

(3) Where claims appear strong (“inhibits,” “therapeutically effective”), rephrase to more cautious language appropriate for computational predictions;

Answer: We have taken the suggestions accordingly.

(4) Include a short limitations paragraph noting the need for biochemical and cellular validation.

These improvements will enhance the manuscript’s clarity and scientific rigor. Overall, the study is well-executed and suitable for publication after minor revision.

Answer: We have included the section in conclusion section.
