# Supplementary material for: Targeting epigenetic regulators: In-silico discovery of natural inhibitors against histone demethylase KDM4C
Source: PLoS One. 2026 Jan 8;21(1):e0340107. doi: 10.1371/journal.pone.0340107 (PMC12782423; doi:10.1371/journal.pone.0340107)
Supplement: Attachment — Submitted filename: Epigenetics reviewer comment.docx [file pone.0340107.s001.docx]

**Reviewer Comment**

The paper titled “Targeting epigenetic regulators: In-silico discovery of natural inhibitors against histone demethylase KDM4C” looks at how to use computer-based methods to find natural compounds that could block KDM4C, an epigenetic regulator linked to several cancers. The study integrates virtual screening, molecular docking, molecular dynamics simulations, and MM-GBSA free energy calculations to evaluate compound binding and stability. The research focuses on a current and significant topic in cancer treatment by suggesting that computer-identified natural compounds, which have good ADMET profiles, could be used as potential epigenetic therapies. Overall, is a relevant and detailed study with translational potential, though some issues related to presentation, clarity, and critical analysis need attention.

The issues are:

1. Fix the typographical inconsistencies (e.g., “pectolaniarin” vs. “pectolinarin”).
2. The work heavily relies on proprietary software (Schrödinger suite), and it does not discuss reproducibility using open-source tools. Consider including brief commentary on reproducibility or comparison with open-source methods such as AutoDock Vina.
3. The discussion overemphasizes docking scores without accompanying in vitro or in vivo validation, which limits the impact. Add a clearer discussion of the limitations of in silico predictions and the need for experimental confirmation.
4. The conclusion largely restates the abstract and lacks critical evaluation of study limitations. Use the conclusion to reflect on the limitations of computational-only studies and propose concrete next steps (e.g., planned wet-lab assays).
5. Maintain uniform formatting, with some journal titles abbreviated and others not. A uniform referencing style is needed.

Overall, this is a solid and methodologically thorough computational study that identifies natural inhibitors of KDM4C with potential anticancer activity. Although the findings show promise, the manuscript could benefit from a stronger emphasis on the limitations of computational predictions and a more critical discussion. I recommend minor revisions to improve clarity, scientific rigor, and presentation.
